# Decision-Making under Ambiguity Is Modulated by Visual Framing, but Not by Motor vs. Non-Motor Context. Experiments and an Information-Theoretic Ambiguity Model

**DOI:** 10.1371/journal.pone.0153179

**Published:** 2016-04-28

**Authors:** Jordi Grau-Moya, Pedro A. Ortega, Daniel A. Braun

**Affiliations:** 1 Max Planck Institute for Biological Cybernetics, Tübingen, Germany; 2 Max Planck Institute for Intelligent Systems, Tübingen, Germany; 3 Graduate Training Center of Neuroscience, Tübingen, Germany; 4 School of Engineering and Applied Sciences, University of Pennsylvania, Philadelphia, PA, United States of America; Technion Israel Institute of Technology, ISRAEL

## Abstract

A number of recent studies have investigated differences in human choice behavior depending on task framing, especially comparing economic decision-making to choice behavior in equivalent sensorimotor tasks. Here we test whether decision-making under ambiguity exhibits effects of task framing in motor vs. non-motor context. In a first experiment, we designed an experience-based urn task with varying degrees of ambiguity and an equivalent motor task where subjects chose between hitting partially occluded targets. In a second experiment, we controlled for the different stimulus design in the two tasks by introducing an urn task with bar stimuli matching those in the motor task. We found ambiguity attitudes to be mainly influenced by stimulus design. In particular, we found that the same subjects tended to be ambiguity-preferring when choosing between ambiguous bar stimuli, but ambiguity-avoiding when choosing between ambiguous urn sample stimuli. In contrast, subjects’ choice pattern was not affected by changing from a target hitting task to a non-motor context when keeping the stimulus design unchanged. In both tasks subjects’ choice behavior was continuously modulated by the degree of ambiguity. We show that this modulation of behavior can be explained by an information-theoretic model of ambiguity that generalizes Bayes-optimal decision-making by combining Bayesian inference with robust decision-making under model uncertainty. Our results demonstrate the benefits of information-theoretic models of decision-making under varying degrees of ambiguity for a given context, but also demonstrate the sensitivity of ambiguity attitudes across contexts that theoretical models struggle to explain.

## Introduction

Should you continue reading this paper? The uncertainty involved in this decision is difficult to quantify. This is in contrast to uncertainties arising for example in dice or roulette games, where the decision-maker has a pretty good idea of the probabilities that are involved, even though individual outcomes cannot be predicted. In the economic literature there is a longstanding debate about known vs. unknown uncertainty [1], sometimes also called risk vs. ambiguity. The question is, whether these two kinds of uncertainty are the same or whether they are processed in a different way by human decision-makers. This question has been famously addressed by Ellsberg in what is now an eponymous experiment [2]. In a simplified version, it requires subjects to choose between a *risky urn* with a known composition of differently colored balls, for example 50 blue balls and 50 red balls, and an *ambiguous urn* with an unknown color composition, for example 100 balls with unknown proportion of blue and red. When setting a prize on drawing a blue ball, most subjects (typically around 70% [3]) prefer drawing from the risky urn, implying the belief that there are more blue balls in the risky urn than in the ambiguous one. The paradox arises when leaving the urns untouched and swapping the prize money. When setting a prize on drawing red, most subjects still prefer drawing from the risky urn, implying the belief that there are more red balls in the risky urn than in the ambiguous one. Crucially, there is no single probability that can represent the two beliefs that there are simultaneously more blue balls and more red balls in the risky urn than in the ambiguous urn. Ever since the experiments of Ellsberg there has been growing evidence, both behaviorally [4, 5] and neurally [6–11], that there are indeed two different kinds of uncertainty considered by humans engaged in economic decision-making. However, it is unclear how ambiguity is modulated by the task context and by framing.

Previous studies have investigated, for example, how decision-making in sensorimotor tasks compares to economic pen-and-paper decision-making. A number of these studies have reported that the human sensorimotor system operates in line with expected utility theory, that is Bayes-optimal decision-making with known probabilities [12–19]. Other studies have shown discrepencies of sensorimotor decision-making with Bayes-optimal decision-making. In particular, Wu et al. [20] have previously compared economic decision-making with an equivalent motor task where participants had to choose between different targets they had to hit. In particular, they investigated a well-known decision-making paradox under risk—the so-called Allais paradox —and its occurrence in the two types of tasks. They found that subjects had different attitudes towards risk in the two tasks but did not investigate the origin of this behavioral difference. Additionally, in their motor task the targets were always fully visible and, therefore, were not subject to ambiguity.

In this study we ask the same question as Wu et al. [20] did for risk in the Allais paradox now for ambiguity in the Ellsberg paradox. We investigate a generalized version of Ellsberg’s paradox in decision-making under ambiguity and test how ambiguity is modulated by task context and framing. Similar to Wu et al. [20], we compare motor and non-motor context. As a motor context we use a target hitting task, as a non-motor context we use an urn task. Additionally, we investigate the effects of visual framing by manipulating the stimulus presentation, in particular the way how uncertainty is visually displayed. Finally, we compare Bayes-optimal expected utility predictions to predictions of an information-theoretic free energy model of decision-making under varying degrees of ambiguity.

## Results

### An information-theoretic model of decision-making under ambiguity

In Ellsberg’s urn experiment subjects have to choose between two options, a risky urn and a fully ambiguous urn. We generalize this paradigm by also including partially ambiguous urns, which can be experimentally achieved for example by revealing samples from the ambiguous urn with unknown ratio. We assume that subjects’ choice between the risky option *x*_risk_ and the ambiguous option *x*_amb_ can be described by a probability distribution *p*(*x*) with *x* ∈ {*x*_amb_, *x*_risk_}, and that subjects have no prior preference between the options, that is *p*_0_(*x*) = 1/2. Each option *x* is characterized by a latent variable *h* corresponding to the ratio of blue and red balls. Each *h* implies a utility *U*(*h*) indicating the expected payoff under *h*. For the risky option *h* is known, for the ambiguous option it is unknown. The decision-maker holds a Bayesian belief *q*(*h*|*x*, *D*) about *h* for option *x* after observing data *D* corresponding for example to the observed samples in the urn experiment. Accordingly, we have the belief *q*(*h*|*x*_amb_, *D*) for the ambiguous option and the belief *q*(*h*|*x*_risk_, *D*) = *δ*(*h* − h*) for the risky option with a ratio h* of red and blue balls.

The crucial point of Ellsberg’s original experiment was to show that standard models of economic decision-making that only care about maximizing expected utility cannot explain subjects’ choice behavior under ambiguity. In our experiment an expected utility maximizer would assign the value *V*_0_ to option *x* according to

$$\begin{array}{c} V_{0}\left(x\right)=E_{q(h|x,D)}\left[U\left(h\right)\right]. \end{array}$$ (1)

A perfect expected utility maximizer chooses the option x* = arg max_*x*_ *V*_0_(*x*) that maximizes the overall expected utility. A more general imperfect expected utility maximizer can be modeled for example by a soft-max decision rule, such that the decision-maker chooses according to hypothesis **H**_1_

$$\begin{array}{c} H_{1}:\;p_{1}\left(x\right)=\frac{e^{\alpha V_{0}\left(x\right)}}{\sum_{x^{\prime }}e^{\alpha V_{0}\left(x^{\prime }\right)}} \end{array}$$ (2)

with the soft-max parameter *α*.

Our alternative hypothesis **H**_2_ is that the decision-maker optimizes a free energy function that trades off utilities against information-theoretic constraints that can be derived from axiomatic principles [21–23]. Such information-theoretic constraints can reflect for example a lack of available information which makes them interesting for modeling ambiguity. Intuitively, such a decision-maker is sensitive to ambiguity by biasing their belief *q* towards best-case or worst-case utilities depending on whether the decision-maker is ambiguity-seeking or ambiguity-averse. Such ambiguity-sensitive decision-makers would assign the value *V*_*β*_(*x*) to option *x*, where

$$\begin{array}{ccc} V_{\beta }\left(x\right) & = & \underset{\tilde{q}\left(h|x,D\right)}{\mathrm{ext}}\left\{E_{\tilde{q}\left(h|x,D\right)}\left[U(h)\right]-\frac{1}{\beta }D_{\text{KL}}\left(\tilde{q}\left(h|x,D\right)||q\left(h|x,D\right)\right)\right\}\\ & = & \frac{1}{\beta }\log E_{q(h|x,D)}\left[e^{\beta U(h)}\right] \end{array}$$ (3)

**Fig 1:**
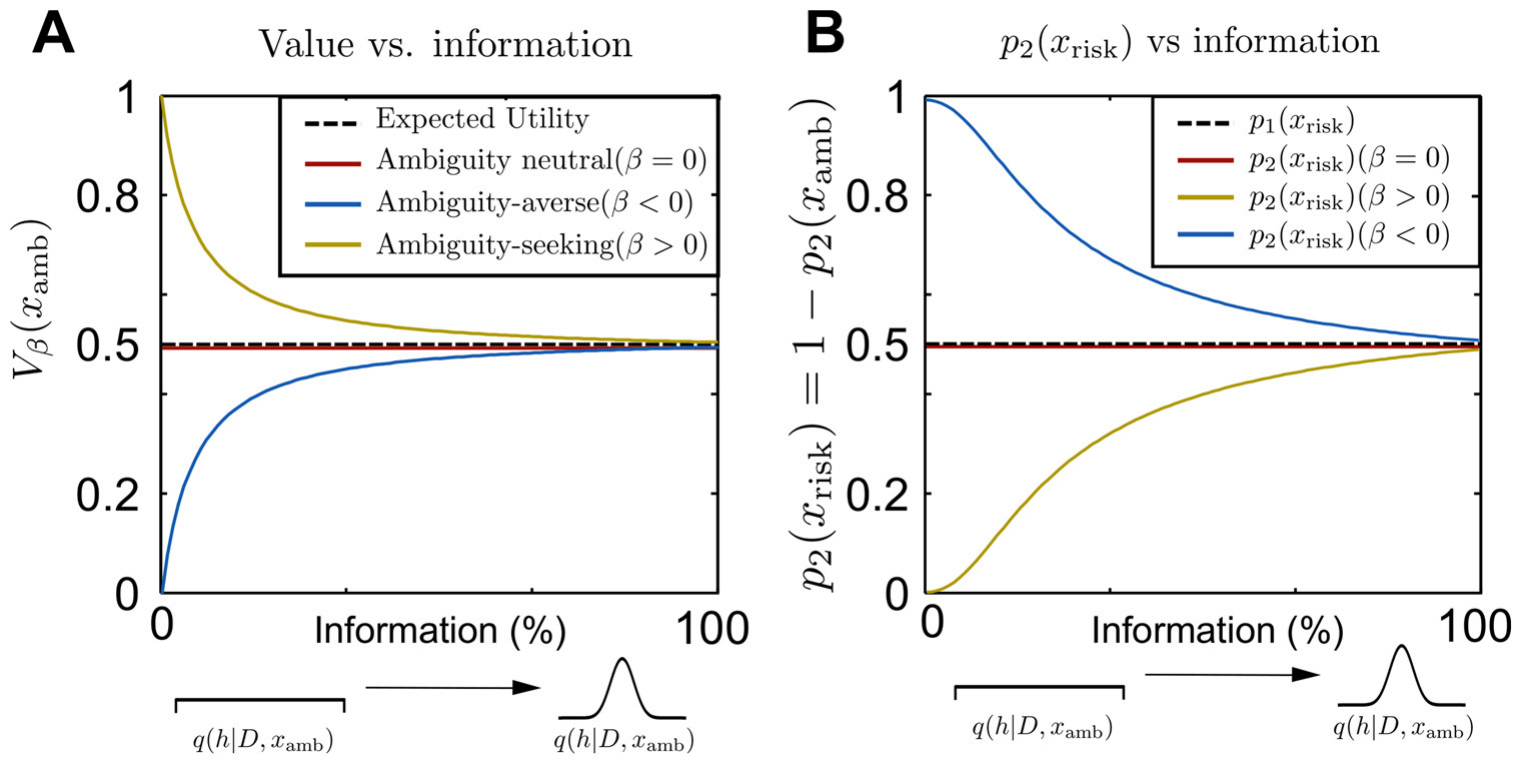
Illustration of model predictions. Predictions for probe trials where the risky urn (with equal composition of blue and red balls) has the same expected utility as the ambiguous urn—the number of observed red balls is equal to the number of observed blue balls. In panel **A** we show the value of Eq (3) assigned to an ambiguous option depending on the ambiguity attitude *β* and on the available information. In the case of the urn, information is quantified with the number of observations. The more information becomes available the more concentrated is the Bayesian posterior *q*(*h*|*D*, *x*), so a high number of observed balls reflect a peaked posterior. We show in yellow line the value of the ambiguous option *V*_*β*_(*x*_amb_) according to Eq (3) for positive *β* (optimistic) being higher or equal than the expected utility value $E_{q|x_{\text{risk}}}=0.5$ (indicated by the dashed line). In blue we show that for negative *β* (pessimistic) the value *V*_*β*_ is always lower or equal than the expected utility value. The value *V*_*β*_ converges to the expected-utility value if the decision-maker is ambiguity-neutral (red line for *β* → 0) or in the absence of ambiguity when the posterior becomes a delta function *q*(*h*|*D*, *x*) = *δ*(*h* − h*). In panel **B** we show the predicted choice probability in probe trials according to Eq (4) for different *β*. Translating the value into a choice probability requires an additional parameter *α* that regulates the level of stochasticity like in a soft-max choice rule. For example, we show in yellow for a particular *α* > 0 how the probability of choosing the risky option is modulated by the information available. The dashed line indicates the perfectly rational expected utility maximizer that is indifferent between the risky and the ambiguous option in the probe trials.

This valuation allows for pessimistic deviations from the Bayesian posterior *q* towards worst-case (ext = min) outcomes if the decision-maker is ambiguity-averse (*β* < 0); or for optimistic deviations towards best-case (ext = max) outcomes if the decision-maker is ambiguity-seeking (*β* > 0). The deviation from the Bayesian posterior *q* is measured by the “information distance” $D_{\text{KL}}(\tilde{q}\left||q\right)$ and scaled by 1/*β*. The larger the magnitude of *β*, the higher the ambiguity regarding *q*. In Fig 1A it can be seen that $V_{\beta }\left(x\right)\;≶\;V_{0}\left(x\right)$ for β ≶ 0. In the economic literature the free energy valuation of Eq (3) is known as multiplier preference models [24] that are part of the more general family of variational preference models [25]. According to [21–23], the decision-maker also optimizes a free energy to determine its action by following the strategy

$$\begin{array}{ccc} H_{2}:\;p_{2}\left(x\right) & = & \underset{\tilde{p}\left(x\right)}{arg max}\left\{E_{\tilde{p}}\left[V_{\beta }\left(x\right)\right]-\frac{1}{\alpha }D_{\text{KL}}\left(\tilde{p}\left(x\right)||p_{0}\left(x\right)\right)\right\}\\ & = & \frac{p_{0}\left(x\right)e^{\alpha V_{\beta }\left(x\right)}}{\sum_{x^{\prime }}p_{0}\left(x^{\prime }\right)e^{\alpha V_{\beta }\left(x^{\prime }\right)}} \end{array}$$ (4)

which is equivalent to a soft-max choice rule when assuming an indifferent prior choice probability of $p_{0}\left(x\right)=\frac{1}{2}$. Such a free energy optimizing decision-maker can be interpreted as a bounded rational decision-maker that can only afford to deviate from the prior choice strategy *p*_0_(*x*) by a limited number of information bits quantified by the relative entropy *D*_KL_(*p*||*p*_0_) [23]. Eq (4) describes the choice of option *x* with value *V*_*β*_(*x*) under both sensitivity to ambiguity and limited information-processing resources—see Fig 1B. Note that the two hypotheses are nested, as **H**_2_ includes **H**_1_ in the limit of *β* → 0 (no sensitivity to ambiguity) and also includes the perfect Bayes-optimal decision-maker for *α* → ∞ and *β* → 0.

To distinguish between the two hypotheses in our experiment we investigate subjects’ choice probabilities in *probe trials* in which a decision-maker that only cares about expected success would be indifferent between the risky and the ambiguous option. The expected utility hypothesis **H**_1_ predicts that subjects should be indifferent between the risky and ambiguous option in probe trials, that is *p*_1_(*x*) = 1/2. In contrast, the free energy hypothesis **H**_2_ predicts that subjects should modulate their choice behavior in probe trials depending on the degree of ambiguity according to Eqs (4) and (3). In particular, ambiguity-averse individuals (*β* < 0) should prefer the risky option in the face of ambiguity, but do so less and less the more information about the ambiguous option becomes available (that is the more concentrated their belief *q*(*h*|*D*, *x*) becomes). Similarly, ambiguity-seeking individuals (*β* > 0) should prefer the ambiguous option, but less and less so with increasing information. These predictions are illustrated in Fig 1. Note that while the model explains choice behavior depending on a given ambiguity attitude *β*, it does not explain how *β* changes across task contexts. Details of the model can be found in the Materials and Methods section.

### Experiments

We designed an experiment to test for differences in choice behavior in motor versus non-motor contexts. Furthermore, we designed a second experiment to control for framing effects that could be induced by the different stimulus designs used in the two tasks. In both experiments subjects had to choose between a risky and an ambiguous option in every trial. The risky option provided full information about the probabilities of the possible outcomes. The ambiguous option was always characterized by a lack of information with respect to the probabilities. We could manipulate the degree of ambiguity by varying the amount of information revealed about the ambiguous option. After the decision was made subjects received a payoff depending on the chosen option.

**Fig 2:**
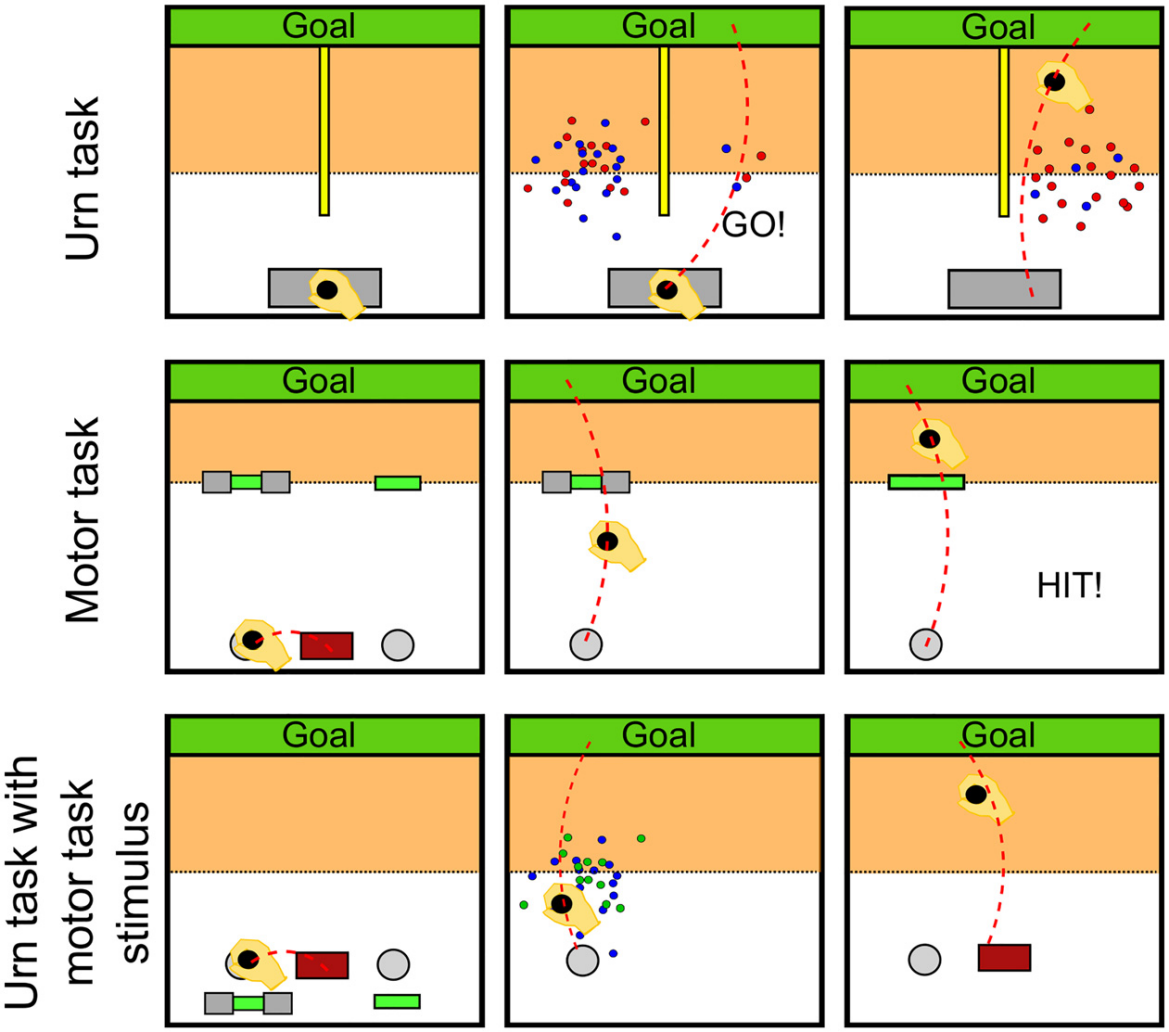
Experimental design. **Top row**. In the urn experiment, the trial was initiated by moving on a gray start bar. Two point clouds appeared showing samples from two urns with different underlying ratios of blue and red balls. The risky urn was always displayed by 100 samples drawn from a 50 : 50 ratio. The ambiguous urn had a variable number of samples drawn from an unknown ratio. Subjects made their decision about the urn they believed to have a higher red ratio by crossing into the area highlighted in orange. In case of choosing the ambiguous urn, the composition of the urn was revealed. The payoff was given by a viscous force field, which was switched on with the probability determined by the chosen urn’s ratio for blue. **Middle row**. In the motor task, subjects had to decide to hit one of two targets by moving into the corresponding decision circle. In case they chose a (partially) occluded target, the target became fully visible after crossing into the highlighted area. When failing to hit the target, subjects had to move against a viscous force field. In both experiments subjects had to move towards the goal bar and back. The orange color is only for illustration and was not displayed during the experiment. **Bottom row**. Experiment 2. Subjects are presented with the same stimulus as in the motor task but perform an urn task where the random outcome is computer generated, in contrast to the motor task where the outcome is determined by the subjects behavior. After choosing between an ambiguous and a risky option a cloud of points appeared revealing the composition of the hidden urn that determined the payoff in the same way as in the urn task.

In Experiment 1 we compare two tasks, an urn task and a sensorimotor task under ambiguity—see Fig 2 top and middle row. In the case of the urn task the stimuli are sampled balls from both urns and the uncertainty about the outcomes after making the choice is computer generated. In case of the motor task, the stimuli are bars that subjects had to hit and therefore the uncertainty about the outcome is internally generated by subjects due to their skill and motor variability. Any difference in behavior between the two tasks might be attributable to either motor vs. non-motor context or to the stimulus design. The goal of Experiment 2 is to distinguish between the two possibilities.

### Experiment 1: Urn task vs motor task

#### Urn task

In the urn task—see Fig 2 top row—the risky option was always fully visible and displayed by a sample of 100 balls drawn from an urn with 50 : 50 composition of red and blue whereas the ambiguous option had a possibly different composition with varying degree of ambiguity depending on the number of samples that were shown, ranging from zero (full ambiguity) to one hundred samples (no ambiguity). Ellsberg’s original task corresponds to the fully ambiguous limit case in which the ambiguous urn shows zero samples.

Subjects decided between the risky and the ambiguous urn displayed in the two halves of the workspace respectively by moving a manipulandum to the respective half—compare Fig 2 top row. To complete the trial they had to move to a goal bar and back to the start position. Instead of a monetary payoff as used in Ellsberg’s original experiment, we used viscous force fields that subjects tried to avoid. The force payoff was stochastic and constituted a risk probability. The probability to experience a force in any individual trial was determined by the probability of drawing a blue ball from the urn chosen by the decision-maker. We recorded subjects’ choice in each trial and determined their choice probabilities as choice frequencies over many trials with the same stimulus. Importantly, we designed symmetric *probe trials* in which half of the shown samples from the ambiguous urn were red and the other half blue, such that the most likely hypothesis to explain this observation is a 50 : 50 composition of red and blue balls. Crucially, subjects should be indifferent between the ambiguous and the risky urn in these trials, if they base their decision solely on their expected success, as posited by expected utility theory given that in our experiment all possible ratios for the ambiguous urn are equiprobable. Additionally, we ascertained subjects’ preference for no-force outcomes in trials without ambiguity (i.e. the ambiguous option was fully revealed), where subjects preferred in more than 93% of cases the urn with the higher ratio of red. In fact, we found the payoff given as a force not to be critical, as subjects in a control experiment that received point scores as payoffs showed the same behavior—compare Figure A in S1 File. This is in line with previous studies [11, 26] that have found ambiguity attitude to be robust in gains vs. losses scenarios.

**Fig 3:**
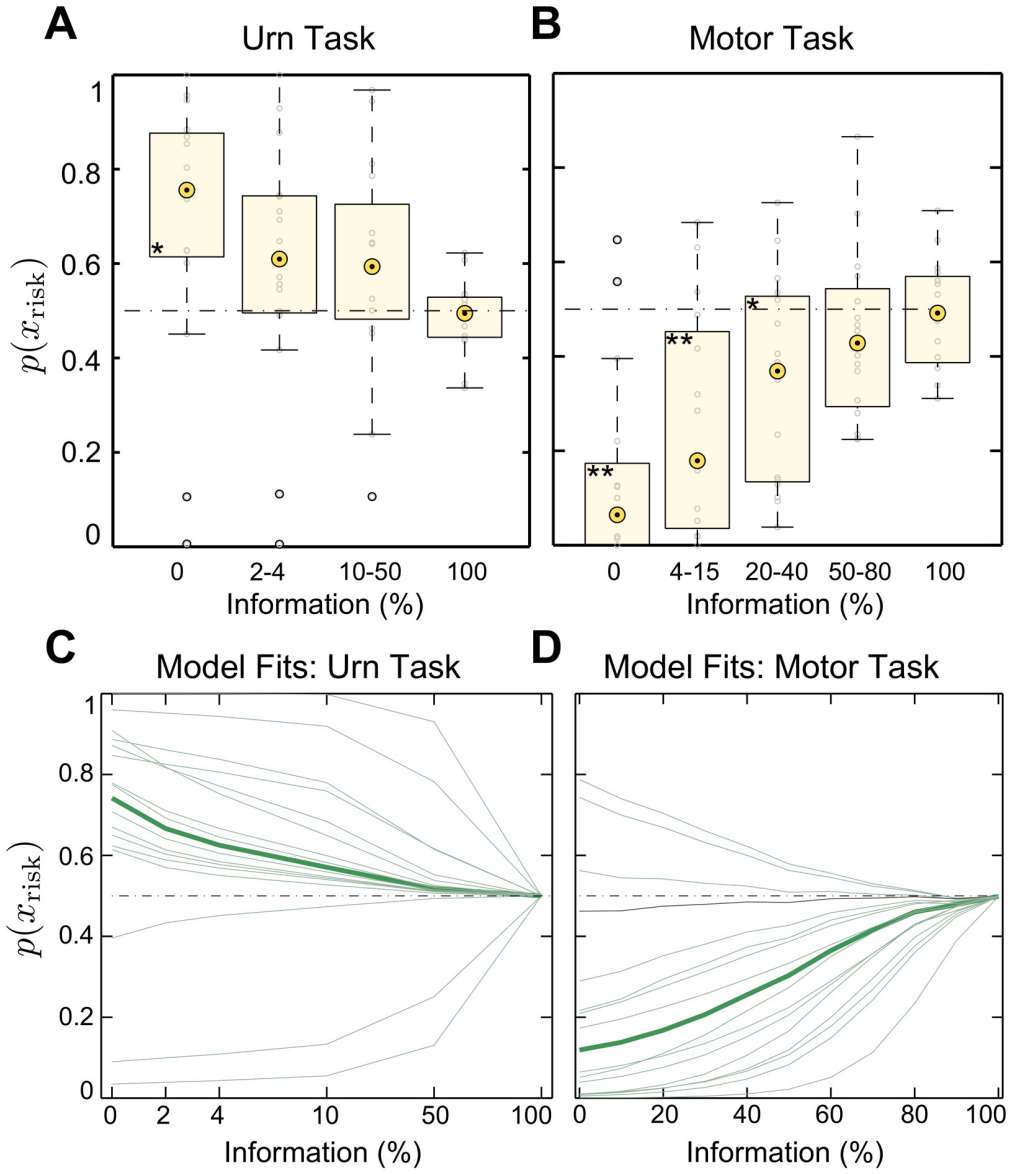
Experiment 1: Experimental data and model fits. Aggregate choice probabilities over all subjects in probe trials of **A** the urn task, **B** the motor task. The boxes are centered around the median across subjects and the edges of the box are the 25th and 75th percentiles. Panels **C** and **D** show the corresponding model fits. The thin green lines represent individual subjects’ choice probabilities according to Eq (4), the thick green line indicates the group mean. The dashed lines show the indifference choice probabilities predicted by expected utility. Probabilities above the dashed line imply that subjects prefer the risky choice (ambiguity aversion), probability values below the dashed line imply that subjects prefer the ambiguous choice (ambiguity preference). Asterisks denote significant deviation from the expected utility prediction: one asterisk signifies *p* < 0.05, two asterisks signify *p* < 0.01. In the urn task information (%) corresponds to the ratio of the number of revealed balls to the total number of balls, in the motor task and in the urn task with motor stimulus to the ratio of visible size to total size of the ambiguous target.

In accordance with Ellsberg’s results, we found in our urn experiment that the majority of subjects were averse to the fully ambiguous urn in the probe trials—see Fig 3A. For 13 out of 16 subjects the choice probability for the risky urn was significantly elevated from 50 : 50 in the fully ambiguous condition (*p* < 0.05, binomial test)—compare Figure B in S1 File for single subject choice data. This deviation from expected utility theory in the zero information limit (full ambiguity) was also significant at the population level (*p* < 0.05, Wilcoxon signed-rank test). In the case of full information (zero ambiguity), the ambiguous urn showed as many samples as the risky urn. Naturally, subjects were indifferent between these two indistinguishable options (*p* > 0.6, Wilcoxon signed-rank test). In between the two information limits, the ambiguous urn was partially revealed by showing a smaller number of samples. We found that subjects’ preference for the risky urn decreased with an increasing amount of information about the ambiguous urn (*p* < 0.05 for 11 out of 16 subjects, Cochran-Armitage trend test with linear weights). Moreover, we found that the time to take the decision increased with an increasing amount of information about the ambiguous urn—compare Figure C in S1 File. Since in the analyzed probe trials we ensured that half of the observed samples were red and the other half blue, a decision-maker that only cares about the expected success would be indifferent between the two options regardless of the amount of information. Such a decision-maker is represented by the dashed flat line in Fig 3A. We found that all but one subject significantly differ from this choice pattern and thereby exhibit ambiguity aversion (13 subjects) or ambiguity-seeking behavior (2 subjects) depending on the degree of available information.

#### Motor task

In the sensorimotor task—see Fig 2 middle row—we translated Ellsberg’s urn task into an equivalent a motor task where subjects had to hit a risky target or an ambiguous target. The risky target was always fully visible, whereas the ambiguous target was occluded in varying degrees, such that subjects could not precisely assess the hitting probability associated with the hidden target size. We manipulated the degree of ambiguity by varying the size of the occluder from no occlusion to full occlusion.

In the motor task subjects chose in every trial between a risky target and an ambiguous target. Once selected, they had to try and hit the target. If they failed to do so, they experienced a viscous force on their way to the goal bar and back. To test the impact of varying ambiguity on choice behavior, we again introduced symmetric *probe trials* in which a decision-maker that only cares about expected success would be indifferent between the risky and the ambiguous option. In the case of the ambiguous target, the size of the occluder and the hidden target size were adjusted in a way such that the expected hitting probability for the subjects in probe trials was also 50%, given that in our experiment all hidden sizes compatible with the occlusion were equiprobable—see Materials and Methods for details. To assess subjects’ hitting probabilities and to adjust the displayed target sizes accordingly, we measured subjects’ endpoint variability and ensured that their performance was stable over at least 500 trials—see Materials and Methods for details. Finally, we ascertained subjects’ preference for no-force outcomes in trials without ambiguity, where subjects preferred the larger target in more than 87% of cases. Again we found the fact that the payoff was given as a force not to be critical, as subjects in a control experiment that received point scores as payoffs showed the same behavior—compare Figure A in S1 File.

In contrast to the expected utility prediction in probe trials that is represented by the dashed lines in Fig 3B, we found that most subjects’ choice probability differed significantly from this prediction, and that consequently their behavior cannot be simply explained by expected utility maximization. However, unlike in the urn probe trials, most subjects had a preference for the ambiguous option in the motor probe trials. When choosing between the risky and the fully ambiguous target—corresponding to Ellsberg’s choice scenario—, 13 out of 16 subjects’ choice probability for the risky target was significantly reduced from 50 : 50 (*p* < 0.01, binomial test)—compare Figure B in S1 File for single subject choice data. This deviation from expected utility theory in the zero information limit was also significant at the population level (*p* < 0.01, Wilcoxon signed-rank test)—compare Fig 3B. In the case of full information (zero ambiguity), both targets were fully visible and indistinguishable (*p* > 0.6, Wilcoxon signed-rank test). In between the two extremes of zero and full information, the ambiguous target was only partially occluded. We found that for 14 out of 16 subjects, preference for the ambiguous target decreased with an increasing amount of information (*p* < 0.05, Cochran-Armitage trend test with linear weights). Unlike in the urn task, we found the decision time in the motor task not to vary with the degree of ambiguity—compare Figure C in S1 File.

#### Model fits

In the information-theoretic free energy model of decision-making there are two free parameters per subject to fit, that are the soft-max parameter *α* and the ambiguity parameter *β*. In contrast, the expected utility model has only one free parameter per subject given by the softmax-parameter *α*. The two decision-making models are nested in the sense that the expected utility model is a special case of the free energy model in the limit *β* → 0. To compare the two hypotheses we maximized the log-likelihood of the experimental data over all trials by varying the free parameters of the two models. We performed a likelihood ratio test to investigate which model fits the data better. Importantly, the likelihood ratio test with nested models trades off the extra complexity of the more general model against its better fitting performance. We found that we can reject the expected utility model with a p-value of *p* < 0.01. The model fits are shown in green in Fig 3C and 3D and in Figure B in S1 File for individual choice data. In Fig 3 it can be seen that unlike the expected utility model, the free energy model can explain how subjects’ choice probabilities change depending on the amount of available information.

While there are a number of alternative ambiguity models, the difficulty in our task is that these models have to be dynamically consistent—that is they have to be updated with new data in a consistent way—and they have to allow for both ambiguity-seeking and ambiguity-averse behavior. For the urn task we adapt one of the most popular ambiguity models from Gilboa and Schmeidler [27], because in this case the prior can be easily parameterized as a beta distribution. The Gilboa-Schmeidler model assumes that decision-makers have multiple beliefs arising from multiple priors. We assume that within that set of priors decision-makers can update their beliefs according to Bayesian inference procedures and select the worst-case possible belief for every option. This will lead to ambiguity averse behavior. To allow for ambiguity-seeking behavior we will also allow for best-case selection of beliefs. We model directly the best- or worst-case belief selection with a single Beta prior for the ratio of the urn. In this case the Gilboa-Schmeidler model has three parameters, the soft-max parameter and two more parameters of the Beta prior. In contrast the information theoretic model has two parameters, the ambiguity parameter *β* and the rationality parameter *α*. We compare these two models using the Bayesian Information Criterion (BIC) and find that the model comparison clearly favors the information-theoretic model (BIC = 8139) over the dynamic Gilboa-Schmeidler model (BIC = 8165), with ΔBIC = 26.

#### Comparison: motor task and urn task

Importantly, both experiments were performed by the same subjects. In total, 11 subjects that were ambiguity averse in the urn task under full ambiguity, preferred the fully ambiguous option in the motor task. Moreover, 2 subjects that were ambiguity averse in the urn task under full ambiguity, were indifferent to full ambiguity in the motor task. Three subjects did not change their preferences across tasks, two of them consistently preferred the fully ambiguous option, one of them remained indifferent. This difference in behavior of subjects between the two tasks might be attributable to either motor vs. non-motor context or to the stimulus design. This is the subject of the second experiment.

### Experiment 2: Stimulus versus motor framing

**Fig 4:**
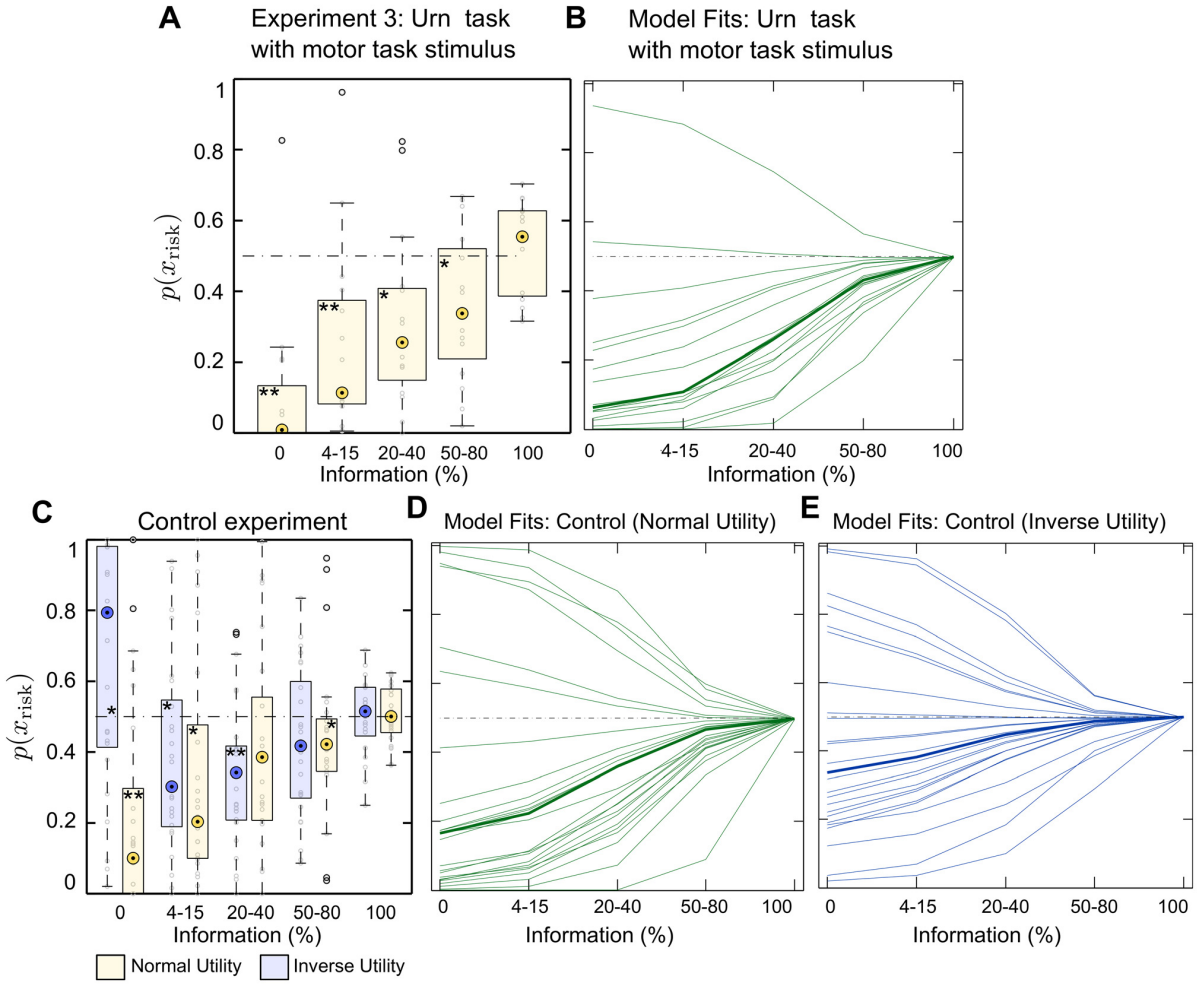
Experiment 2. Panel **A** shows Experiment 3 choice data where subjects are ambiguity-seeking as in the motor task and not ambiguity-averse as in the urn task—compare Fig 3. Panel **C** shows experimental choice data of the control experiment where subjects are ambiguity-seeking in most trials independent of the utility function—normal utility condition as in Experiment 3 or inverse utility condition. Probability values above the dashed line predicted by expected utility imply that subjects prefer the risky choice (ambiguity aversion), probability values below the dashed line imply that subjects prefer the ambiguous choice (ambiguity preference). The normal utility condition is colored in light orange, the inverse utility condition is colored in light blue. The boxes are centered around the median across subjects and the edges of the box are the 25th and 75th percentiles. Asterisks denote significant deviation from the expected utility prediction: one asterisk signifies *p* < 0.05, two asterisks signify *p* < 0.01. Panels **B**, **D** and **E** show the corresponding model fits. The thin lines represent individual subjects’ choice probabilities according to Eq (4), the thick lines indicate the group mean. Note in panel **E**, how the our information-theoretic model (and the expected utility model) is unable to produce simultaneously ambiguity aversion in the zero information limit and ambiguity seeking behavior in the other information cases.

In the first experiment we found a clear difference in choice behavior between the motor task and the urn task—compare Fig 3. This difference in subjects’ behaviour between the two tasks might be attributable to either motor vs. non-motor context or to the stimulus design. In Experiment 2, we distinguish between the two possibilities. In this experiment, a group of subjects performed the urn task but at the moment of choice they were presented with the motor task stimulus instead of the urn task stimulus. If the preference reversal was mainly induced by the stimulus, we would expect most subjects to prefer the ambiguous option in the probe trials of Experiment 2, as the stimulus is identical to the motor task. However, if the preference reversal was mainly a function of the underlying source of uncertainty [28] (external source for the urn task or internal source for the motor task), we would expect them to be mostly ambiguity averse as in the urn task. We found that most subjects in Experiment 2 still preferred the ambiguous option as in the motor task—compare Fig 4A. This deviation from expected utility theory was significant both at the population level (*p* < 0.05, Wilcoxon signed-rank test on the full ambiguity condition) and at the level of individual choice: for 14 out of 16 subjects in Experiment 2, preference for the ambiguous target decreased with an increasing amount of information as in the motor task (*p* < 0.05, Cochran-Armitage trend test with linear weights). This suggests that subjects’ ambiguity preference critically depends on the stimulus display, whereas the context of motor and non-motor task and the framing of gains and losses do not seem to be critical.

We conducted a control experiment to discern if the stimulus affects directly ambiguity attitude or whether it induces a perceptual distortion in such a way that after all subjects’ behavior can be explained according to expected utility with perceptual bias. Importantly, both the control experiment and Experiment 2 were performed by the same subjects. The control experiment was identical to Experiment 2 except that the force payoff was now associated with the opposite color (inverse utility condition). Effectively, this implied that now smaller bar stimuli were preferable to larger bar stimuli. We can then compare subjects’ choices when presented with the same target bar stimulus under the two utility conditions. If they prefer the same option—either ambiguous or risky—under both conditions, their choice cannot be explained by a single belief probability, as they would effectively believe the same stimulus to be larger and smaller at the same time. Crucially, this could be explained in terms of ambiguity attitude. If, however, they believed the ambiguous target to be smaller in one utility condition, but larger in the other, then their behavior might also be explicable as a perceptual bias or a biased belief that discards experienced statistics.

Fig 4C shows subjects’ choice behavior under the two utility conditions. For the normal utility condition (light orange boxes), we see the gradual increase in ambiguity preference as in the previous subject groups. However, for the inverse utility condition (blue boxes) the results are mixed. For partially occluded stimuli subjects still mostly prefer the ambiguous option, but in the case of fully occluded stimuli they mostly prefer the risky option—compare Fig 4C. The corresponding significance values (Wilcoxon signed-rank test) are indicated by asterisks in the figure. This mixed behavior is also visible in single subject choice data—compare Figure D in S1 File. For both, the Experiment 2 with normal utility condition and the inverse utility condition we found the decision time not to vary with the degree of ambiguity—compare Figure C in S1 File. In Fig 4B, 4D and 4E we show the model fits according to Eq (4). In particular, we observe the limitations of the information-theoretic model and the expected utility model when modeling the inverse utility condition experiment (Fig 4E). None of them is able to show both ambiguity averse behavior in the zero-information limit and ambiguity seeking behavior in the remaining ambiguity levels.

A possible reason for the deviation observed in the full ambiguity condition might be the nonlinear relationship between bar size and hitting probability, which only plays a minor role in the partial ambiguity condition. Thus, these mixed results suggest that both perceptual distortion and stimulus-dependent ambiguity attitude play a role in sensorimotor choices. Crucially, it is impossible to exclusively explain the observed preference reversal within expected utility theory with biased beliefs or by perceptual distortion, as it is impossible to probabilistically represent the belief or the perception that the same stimulus is at the same time smaller and bigger than a gauge stimulus, as observed in the partial ambiguity conditions.

## Discussion

In our study we found that human subjects continuously modulate their choice behavior in an experience-based urn task and in a motor task depending on the level of ambiguity in line with the prediction of a information-theoretic free energy choice model and contrary to the prediction of expected utility. We found that the ambiguity preference changed in the two tasks for the same subjects, where subjects were mostly ambiguity-averse in the urn task and ambiguity-seeking in the motor task. Additionally, we found that subjects’ ambiguity sensitivity is not affected by the framing of motor and non-motor context. However, in a second experiment we show that this reversal was mainly a consequence of a framing effect induced by the different stimuli in the two tasks.

Framing effects induced by presenting decision-problems in terms of gains and losses were first studied by Tversky and Kahneman [29], showing how framing could greatly affect choice behavior. In our study we found that the framing effect induced by stimulus display significantly affected behavior. In fact, the visualization of uncertainty has recently become an active research topic [30–33]. One of the reasons for this surge in interest is the realization that the way uncertainty is communicated can affect policy making, for example in the context of the climate change debate. Similarly, our results suggest that the way that ambiguity is presented to users can make striking differences in the way they respond to this uncertainty, both in economic decision-making tasks and in motor tasks. A previous study by Wu et al [20] have also reported striking differences between economic and sensorimotor decision-making under risk. Our results suggest that these differences could be explained by the way uncertainty is displayed and not by the fact of how uncertainty is generated—externally in case of economic task or internally in case of a sensorimotor task. In principle, the representation of uncertainty can induce both perceptual biases or elicit particular ambiguity attitudes. In our tasks, we found that perceptual biases alone cannot explain subjects’ choice behavior and that ambiguity attitude is affected in stimuli with partial ambiguity.

Previous studies in behavioral economics have shown that risk-attitudes can be distinguished experimentally from ambiguity attitudes [5]. Risk attitudes are usually modeled by the curvature of the utility function [34, 35]. This model of risk is also included in Eq (3). The ambiguity attitude in the free energy model is expressed by an additional temperature parameter that quantifies deviations from a Bayesian model [23]. The same variational principle can also be applied to acting of bounded rational decision-makers. In this case, the temperature parameter *β* can be interpreted in terms of the degree of control a decision-maker has as a result of the available computational resources. Accordingly, one could interpret Eq (3) equivalently as anticipating the choice of a bounded rational opponent with boundedness parameter *β*. Therefore, our results encourage a more general investigation of free energy variational principles for perception and action. One such avenue might be the study of decision-makers’ perceived degree of control, for example in the context of illusions of control [36, 37]. In the case where utilities are restricted to informational surprise or absorbed into prior distributions, such free energy variational principles have for example been recently investigated by Friston and colleagues [38, 39].

In the economic literature there have been an extensive effort in developing models that formalize decision-making under ambiguity. From the first models where decisions are evaluated by looking exclusively at its worst possible outcome [40], to models that take into account both the worst and the best possible outcome [41]. There are also more mathematically elaborate models such as Choquet Expected Utility (CEU) model [42] where beliefs are not considered subjective probabilities but by capacities that can possibly be non-additive. Extensions of CEU include the Cumulative Prospect Theory [43] that uses two capacities, one for gains an another one for losses. There are other popular models such as the Maxmin expected utility model from Gilboa and Schmeidler that use multiple priors to define the beliefs of decision-makers with built-in ambiguity aversion [27] and also a variation of it that drops the axiom of ambiguity aversion [44]. The smooth ambiguity aversion model [45] can be viewed as an extension of the maxmin model. It regards the maxmin criterion as too extreme and opts for modeling second order beliefs and introducing a convex function to model ambiguity aversion—in the same way that the curvature of the utility function models risk aversion.

The information-theoretic model relates to the above-mentioned models in several ways. First, Eq (3) that assigns value to an option under ambiguity presented here is known in the economic literature as a multiplier preference model [24], that is a type of variational preference model for decision-making under ambiguity [25, 46]. In our formalism, the temperature parameter can assume positive and negative values corresponding to ambiguity-seeking or ambiguity-averse behavior without changing the general form of the solution equations. Second, just like the multiplier preference model the information-theoretic model has dynamic consistency, because it can incorporate new information in line with Bayesian updating [47]. Accordingly, it provides a neat way to include ambiguity into the Bayesian formalism, unlike many other models that abandon the concept of Bayesian probability. Third, the majority of the decision-making models under ambiguity include an *ad hoc* soft-max function to determine the probabilities of decisions. In contrast to these previous models, we use a single free energy principle for both action (Eq (4)) and perception (Eq (3)) which can also be reconciled with Bayesian updating (that also obeys a free energy principle) and dynamic choice under new incoming data [48]. Therefore, the information-theoretic model provides a powerful generalization to Bayes optimal decision-making allowing for ambiguity and limited resources.

Variational ambiguity models build on earlier work on robust control where decision-makers consider the possibility that their current model *q* may not be the appropriate model for the observed phenomenon and therefore bias their predictions towards worst-case outcomes to ensure robustness [24]. The concept of robustness is also closely related to the concept of risk-sensitivity as the relative entropy contains the information of all the higher-order statistical moments. Previously, risk-sensitivity was shown to play an important role in motor tasks, showing that subjects care about higher order moments of the cost function. This is often modeled as a mean-variance trade-off, that can be used to express risk attitudes towards observable random variables [49–51]. Previously, it was also shown that risk-sensitivity affects sensorimotor integration when different beliefs are associated with different sensorimotor costs [52] and it also affects the amount of cooperation in two-player games when different beliefs represent the strategy of the other player [53]. In this study we show that the same framework that is used to model risk-sensitivity can also be applied to model ambiguity.

Bayes optimal decision-making has been applied as a very general optimality principle to explain behavior from the scale of single neurons [54, 55] to whole-body motor control [56]. When probability models are accurate, optimal decision-making is indeed optimal and accomplished by computing expected utilities. However, if probability models are inaccurate or even plain wrong, then maximizing expected utility can be far from optimal. In such scenarios, one might be interested in robust control and decision-making strategies with guaranteed performance bounds within defined neighborhoods of a proposed model [24]. Robustness is also a core feature of biological organisms coping with model uncertainty [57], which has so far been neglected in many optimality models. Our results suggests a way of how to combine model uncertainty, optimality and inference in the study of adaptive behavior.

## Materials and Methods

### Ethics Statement

The study was approved by the ethics committee of the Max Planck Society (reference number: 0269/2010BO2). All participants gave written informed consent.

### Subjects

68 subjects (30 male, 38 female) from the Tübingen University student population participated in this experiment after giving informed consent. We excluded one subject in the motor task with force payoffs and two subjects in the motor task with point payoffs, because the standard deviation did not stabilize over the course of the experiment. The remaining 65 subjects were assigned as follows to the three experiments: 16 subjects participated in Experiment 1, 16 subjects participated in Experiment 2, and in Experiment 2 there were 16 subjects in the main experiment (8 of which overlapped with 8 subjects from Experiment 1), and 25 subjects in the control. Participants were paid the local standard rate of 8 Euros per hour for their participation.

### Materials

The experiments were conducted using a vBOT robotic manipulandum [58]. Participants controlled the vBOT handle in the horizontal plane. Movement position and velocity were recorded at a rate of 1*kHz*. A planar virtual reality projection system was used to overlay images into the plane of movement of the vBOT handle. Subjects hand position was displayed by a cursor that could move across the planar screen. Subjects were using their preferred hand throughout the entire experiment.

### Information-theoretic model details

In our experiment decision-makers have ambiguity about a latent variable *h*, which is the unknown ratio of blue and red balls in case of the urn, or the size of the hidden target in case of the motor task. The expected utility for a known *h* is determined by *U*(*h*) = ∑_*o*_ *p*(*o*|*h*)*r*(*o*), where *r*(*o*) = −1 is the reward for the outcome *o* = *blue* in the urn task or *o* = *fail* in the motor task, and *r*(*o*) = 0 for the outcomes *o* = *red* or *o* = *hit*. *p*(*o*|*h*) indicates the probability of drawing color *o* from an urn with known ratio *h* or the probability of hitting a fully visible target of known size *h* depending, of course, on subjects’ skill level. Note that we took into account changes in subjects’ performance during the whole experiment—see *Experimental design: motor task* in the Materials and Methods section.

The decision-maker’s model *q* is given by a Bayesian posterior *q*(*h*|*D*, *x*) over the latent variable *h* when observing data *D* of option *x*. In the urn task, the data corresponds to the number of observed red and blue balls and the distribution *q*(*h*|*D*, *x*_amb_) can be represented by a Beta distribution over the ratio of the ambiguous urn. In the motor task, the data corresponds to observing the occluded target, where *q*(*h*|*D*, *x*_amb_) is a uniform distribution over the possible target sizes covered by the occluder as we sampled the target sizes from this uniform distribution.

The critical trials for model comparison are the probe trials, in which the expected utility of the ambiguous option is exactly the same as the expected utility of the risky option. Importantly, a pure expected utility decision-maker with *β* = 0 values the ambiguous option *x*_amb_ according to its expected utility *V*_0_(*x*_amb_) = ∫*dhq*_amb_(*h*|*D*, *x*_amb_)*U*(*h*), which in the illustration in Fig 1 simply corresponds to the mean of the distribution *q*(*h*|*D*, *x*_amb_). In probe trials the mean is given by $E_{q(h|D,x)}\left[U\left(h\right)\right]=1/2$ by design. Crucially, in probe trials the expected utility value is independent of the number of observed data points. In contrast, the valuation given by Eq (3) is sensitive to the number of data points that determine the spread of the distribution *q*(*h*|*D*, *x*). The more data becomes available the more concentrated the posterior becomes around the true value h* (that is the true ratio of the ambiguous urn or the true target size of the ambiguous target). In the limit of exact knowledge only the true value h* has non-zero probability mass, that is *q*(*h*|*x*, *D*) → *δ*(*h* − h*). In the limit of infinite data, all ambiguity vanishes and the value of the ambiguous option according to Eq (3) converges to *V*_*β*_(*x*_amb_) → *U*(h*) independent of the value of *β*. The limit value *U*(h*) exactly corresponds to the value *V*_0_(*x*_risk_) of the risky option in probe trials—compare Fig 1.

The solution to Eq (4) that describes the choice probability of subjects choosing the risky option is given by

$$\begin{array}{ccc} p_{2}\left(x_{\text{risk}}\right) & = & \frac{p_{0}\left(x_{\text{risk}}\right)e^{\alpha V_{\beta }\left(x_{\text{risk}}\right)}}{p_{0}\left(x_{\text{risk}}\right)e^{\alpha V_{\beta }\left(x_{\text{risk}}\right)}+p_{0}\left(x_{\text{amb}}\right)e^{\alpha V_{\beta }\left(x_{\text{amb}}\right)}} \end{array}$$

where *p*_0_(*x*_risk_) = *p*_0_(*x*_amb_) = 1/2. Naturally, the probability of choosing the ambiguous option is modeled by *p*(*x*_amb_) = 1−*p*(*x*_risk_). Note that the case of uniform prior the choice probabilities follow the common soft-max rule but for non-uniform prior it is a weighted version of this rule.

The value *V*_*β*_(*x*) is the solution to Eq (3) and is given by

$$\begin{array}{c} V_{\beta }\left(x\right)=\frac{1}{\beta }\log Z_{\beta }\left(x\right)=\frac{1}{\beta }\log\int q\left(h|D,x\right)e^{\beta U(h)}dh. \end{array}$$

As the utility function *U*(*h*) and the Bayesian posterior *q*(*h*|*D*, *x*) are given by our modeling assumptions, there are only two free parameters per subject to fit in the information-theoretic free energy model of decision-making, that are the soft-max parameter *α* and the ambiguity parameter *β*.

### Experimental design: Experiment 1 (urn task)

#### Experiment

Subjects performed 600 trials of reaching movements from a start bar (gray rectangle with size 4 × 1.5*cm*) to a goal bar (green rectangle with size 20 × 0.5*cm*) that was 24*cm* away by moving a cursor (red circle, radius 0.3*cm*) representing their hand position—compare Fig 2. After holding still for 0.2*s* at the start bar, subjects heard a beep indicating trial start and stimulus appearance. By moving to the left or right side of the workspace when entering the force zone at 12*cm* in the forward direction (orange zone in Fig 2), they made a choice between the “risky” urn and the “ambiguous” urn. The display location of the risky and the ambiguous urn was randomly selected between left and right. The choice had to be made within a maximum time window of 1*s* after stimulus appearance, otherwise a new trial was generated.

Subjects were informed that both urns contained 100 balls. Subjects were also told that the risky urn always had 50 blue balls and 50 red balls and that the ambiguous urn had an unknown proportion of red and blue balls. Before they had to make their decision they were shown a sample of 100 balls drawn with replacement from the risky urn and a sample of varying size from the ambiguous urn. The number of samples shown from the ambiguous urn was determined randomly from the set {0, 2, 4, 10, 50, 100}. Thus, showing 0 balls corresponds to a completely ambiguous urn and showing 100 balls corresponds to a non-ambiguous urn. We devised two methods to indicate the missing information to ensure robustness of our results. The first eight subjects were explicitly told that the ambiguous urn had 100 balls with only a small subset shown as a sample. The second eight subjects were shown gray balls in ambiguous trials to visualize the missing information directly. Samples from the ambiguous urn were generated as follows. In 50% of trials the composition of the ambiguous urn was determined randomly from the set {(0, 100), (10, 90), …(50, 50), …(90, 10), (100, 0)} of red balls and blue balls respectively. The specified amount of samples was then drawn from the ambiguous urn. In the other 50% of trials, we designed *probe trials* where a perfectly symmetric stimulus was presented where half of the samples was red and the other half was blue. These *probe trials* are important for the model comparison.

To show subjects the samples stemming from either urn, circles of red and blue colors (radius 2*mm*) were drawn from a two-dimensional Gaussian distribution with mean *μ*_*left*_ = −5.0*cm* *μ*_*right*_ = 5.0*cm* and standard deviation *σ*_*left*_ = *σ*_*right*_ = 1.0*cm*. The circles were displayed in the horizontal plane as illustrated in Fig 2. In case subjects opted for the ambiguous urn, the content of the urn was revealed after their choice in order to provide them with feedback. In probe trials, the feedback was given by a sample of 100 balls drawn from a 50 : 50 urn. In other trials, the 100 balls sample was drawn from the ambiguous urn with the specified composition.

Subjects were told to imagine that a ball would be randomly drawn from the urn that they chose, and if the ball was blue they would experience a viscous force *F* in the forward-backward direction when trying to reach the goal bar. The force was set to *F* = −*kv*_*y*_ with $k=1.25\frac{Kg}{s}$ and *v*_*y*_ the velocity of the robot handle in the forward-backward direction. In contrast, if the ball was red subjects would experience no force. The constant *k* of the viscous force was ramped up from *k* = 0 to *k* = 1.25 in the first third of the force area (12*cm* − 16*cm*) in the forward movement and similarly was ramped down in the backward movement to have a smooth transition between the non-force area and the force area.

#### Sampling procedure

In the urn task, the manipulation of the degree of ambiguity was controlled by the number of samples shown from the ambiguous urn. For finite size urns, the problem of inferring the true ratio of red and blue balls of the ambiguous urn depends in general on whether assuming a sampling scheme with replacement or without replacement. While subjects could in principle use either inference strategy, importantly this does not affect our conclusions. In the critical trials with symmetric evidence (probe trials), the expected utility of the ambiguous option is exactly the same under both sampling schemes and equal to the expected utility of the risky option, because in this case the mean of the beta distribution (inference with replacement) is equal to the mean of a beta-binomial distribution (inference without replacement). Our results are consequently independent of the sampling scheme that subjects were using for inference.

### Experimental design: Experiment 1 (motor task)

#### Experiment

Subjects had to move a cursor to a red start rectangle that was placed in the bottom middle of the workspace—compare Fig 2. When subjects entered the red rectangle, two decision circles (radius 0.6*cm*) appeared to the left (−4*cm*) and to the right (4*cm*) of the center of the start rectangle together with their respective targets, and the red bar disappeared. One decision circle was associated with the risky target, while the other one was associated with the ambiguous target. The targets were displayed 18*cm* in the forward direction from the decision circles. The location of the risky target and the ambiguous target was randomized between left and right with 50 : 50 probability. Subjects could compare the two targets and move towards the decision circle associated with the target that they intended to hit. When holding still in the decision circle, the other decision circle and target disappeared and they heard a beep that urged them to move towards the target they selected. In order to increase the difficulty of the target hitting task, we imposed a lateral gain *g* = 3 between hand and cursor movement, thereby artificially increasing the variance of subjects’ reaching endpoints. When hitting the target, they heard a high frequency beep. When missing the target, they heard a low frequency beep. In the latter case, they also experienced a viscous force *F* impeding their movement in the forward-backward direction between the target and a goal bar (between 18*cm* and 27*cm* from the decision circle) they had to reach to complete the trial. The viscous force *F* = −*kv*_*y*_ was proportional to subjects’ movement velocity. To provide a smooth transition between the non-force area and the force area, *k* was ramped up in the forward direction from *k* = 0 to $k^{*}=0.6\frac{Kg}{s}$ within the first quarter between the target and the goal bar, and similarly, ramped down in the backward direction. When subjects reached the goal bar, they heard another beep with the same frequency as before to inform them that the trial was completed. At this point they had to move back to the red rectangle to initiate the next trial. Each trial had to be completed within 0.6*s*.

In case of a fully visible target with half-width *s*, the probability of hitting the target *P*_*hit*_ can be computed as

$$\begin{array}{c} P_{hit}\left(s\right)=2\left(F\left(s;\sigma_{0}^{2}\right)-F\left(0;\sigma_{0}^{2}\right)\right), \end{array}$$ (5)

assuming that subjects’ reaching endpoints can be described by a zero-mean Gaussian distribution with variance $\sigma_{0}^{2}$ such that $F\left(x;\sigma_{0}^{2}\right)=\int_{-\infty }^{x}N\left(x;0,\sigma_{0}^{2}\right)dx$. In case of an ambiguous target with visible size 2*s* and gray occluders of size *d* on each side, the average hitting probability is

$$\begin{array}{c} P_{hit}\left(s,d\right)=\frac{2}{d}\int_{s}^{s+d}\left(F\left(x;\sigma_{0}^{2}\right)-F\left(0;\sigma_{0}^{2}\right)\right)dx, \end{array}$$ (6)

assuming that all possible target sizes are equally probable.

#### Training and tracking performance

At the beginning of the experiment subjects were exposed to a training session where they had to hit a single fully visible target (width 2*cm*) displayed randomly at the left or right target position. After 200 trials, the training session ended allowing us to estimate subjects’ hitting accuracy. In particular, we could compute the target half-width s*, such that subjects’ hitting probability was *P*_*hit*_(s*) = 0.5. To determine s* we computed the median of subjects’ unsigned endpoints. In order to keep track with potential changes in performance after the training session, we continuously adapted s* and all other target sizes based on the penultimate 200 trials ensuring that subjects keep a constant performance over the entire course of the experiment. In total, subjects performed at least 750 choice trials under the condition that the relative standard deviation of s* lies within a band of 10% over the last 500 trials.

#### Trial generation

Ambiguous trials were generated in the following way. Analogous to our urn experiment, in 50% of the choice trials the hitting probability of the ambiguous target was set to *P*_*hit*_ = 0.5. In the other 50% of trials the ambiguous target was set to have a hitting probability drawn randomly from *P*_*hit*_ ∈ {0.3, 0.4, 0.5, 0.6, 0.7}. Larger hitting probabilities were not considered because of the disproportionate target sizes required. Once *P*_*hit*_ was determined, the maximum ambiguous size *d*_*f*_ was computed according to Eq (6) for *s* = 0. Then an ambiguity index *a* was drawn randomly from the set {0, 0.1…0.5…0.9, 1} and the actual size of the occluders was computed as *d* = *ad*_*f*_. This way it could be ensured that all degrees of ambiguity were equally probable. Finally, given the expected hitting probability *P*_*hit*_ and the occluder size *d*, the displayed target half-width *s* was chosen to satisfy Eq (6).

### Experimental design: Experiment 2

In the second experiment subjects were shown the same stimulus as in the motor task, but then experienced the an externally imposed uncertainty as in the urn task. Instead of red and blue, the urn stimulus consisted of green and blue balls to match the color of the target and to establish an association between target size and ratio. The ratio of blue and green balls in the urn was determined by the hitting probability of the true target size under a variance of 1.44*cm*^2^. Subjects initiated each trial by moving their cursor to a red start rectangle as in the sensorimotor experiment. Then two decision circles (radius 0.6*cm*) appeared to the left (−4*cm*) and to the right (4*cm*) of the center of the start rectangle and the two respective target stimuli—one risky, one ambiguous—were displayed 2*cm* below the decision circles. Once the target was selected, a cloud of points was shown 4*cm* above the decision circle to represent the urn. Once the goal bar was crossed the red start bar reappeared so that subjects could trigger the next trial.

#### Main experiment

In each trial of the Experiment 2, subjects chose between a risky target and an ambiguous target with the same statistics as in the motor task, including the occurrence of probe trials with equal expected utility for both options. Once subjects made their choice based on the target stimulus by moving the cursor into one of the decision circles, a cloud of points appeared as in the urn task representing a sample of blue and green balls (instead of red), as subjects were told that the size of the green target bar indicated the ratio of green balls in the urn. As in the urn task, this ratio also determined the probability of the force payoff.

We recorded eight subjects in this control experiment after they performed in the motor task, and another eight control subjects after they performed in the urn task to account for order effects. Unlike the first group, the second group of eight subjects were not yet acquainted with the bar stimulus once they started the control experiment. We therefore adapted the control experiment for them in such a way that they could learn the relationship between ambiguous target stimuli and true target size. Once they selected the target, the true target size was revealed at the same time as showing the true composition of the urn. In contrast, the first eight subjects already knew the bar stimulus from the preceding motor task. Once they selected the target in the control task, they were shown a point cloud that consisted of 100 × *a* gray balls determined by the ambiguity index *a* associated with the ambiguous target and a sample of 100 × (1−*a*) green and blue balls drawn from the corresponding urn with composition equal to *P*_*hit*_. The true composition of the urn was revealed when entering the force zone as in the urn task. This way they could learn a direct mapping from ambiguous bar stimulus to ambiguous urn stimulus. The remainder of the trial proceeded for both control groups as in the urn task. There was no qualitative difference between both groups, as both predominantly preferred the ambiguous option. Accordingly, we found that the preference of subjects first performing in the motor task remained stable across tasks, but the preference of subjects first performing in the urn task changed—compare Figure E in S1 File.

#### Control experiment

In this control experiment we tested a group of subjects performing both in the normal and in an inverted utility condition using the same design as in Experiment 2. In the normal utility condition, a force payoff was associated with drawing a blue ball from the associated urn as in the previous experiment (Experiment 2). In the inverse utility condition, a force payoff was associated with drawing a green ball (instead of red) from the urn. Using the sensorimotor stimulus, effectively, subjects had to decide in the first condition which of the two target bars—risky or ambiguous—they believed to be larger, whereas in the second condition they had to decide which one they believed to be smaller.

In the inverse utility control experiment, sixteen subjects performed the inverse utility condition before performing in the normal utility condition, and nine subjects performed the inverse utility condition after performing in the normal condition. Subjects were told that in both conditions the size of the green bar indicated the proportion of green balls in the urn and that green balls would either be associated with no force (normal condition) or with a force (inverse condition) according to the probability of drawing a green ball from the urn. Since these subjects did not previously perform in the motor task, they underwent the same procedure as the second group of eight subjects in Experiment 2.

#### Order effects

In all experiments, the order in which subjects performed the experiments was permuted. From the sixteen subjects performing the urn and motor task, the first eight subjects started with the urn task, while the second eight subjects started with the motor task. Before performing Experiment 2, the first eight subjects of Experiment 2 performed the motor task and the second eight subjects performed the urn task. In the control experiment, the first sixteen subjects performed the inverse utility condition before the normal condition, and nine subjects performed the normal condition before the inverse condition. To test for order effects we devised both a logistic generalized linear mixed model that depended on an order variable and another logistic generalized linear mixed model that did not depend on it. In both models the other fixed effects were given by the ambiguity condition and the expected hitting probability. The random effect in both models was given by the subject index. We found that in none of the above cases the order played a significant role (*p* > 0.05 in all cases, *χ*^2^ difference test).

## Supporting Information

S1 File Supplementary Figures. (PDF)

S2 File Data. (ZIP)
