# Supplementary material for: Decision-Making under Ambiguity Is Modulated by Visual Framing, but Not by Motor vs. Non-Motor Context. Experiments and an Information-Theoretic Ambiguity Model
Source: PLoS One. 2016 Apr 28;11(4):e0153179. doi: 10.1371/journal.pone.0153179 (PMC4849728; doi:10.1371/journal.pone.0153179)
Supplement: S1 File — (PDF) [file pone.0153179.s001.pdf]

Supplementary Information: Decision-making under ambiguity is modulated by visual framing, but not by motor vs. non-motor context. Experiments and an information-theoretic ambiguity model.

Jordi Grau-Moya[1,2,3,*], Pedro A. Ortega[4], Daniel A. Braun[1,2],

1 Max Planck Institute for Biological Cybernetics, Tübingen, Germany
2 Max Planck Institute for Intelligent Systems, Tübingen, Germany
3 Graduate Training Center of Neuroscience, Tübingen, Germany
4 School of Engineering and Applied Sciences, University of Pennsylvania, Philadelphia PA, USA

*** jordi.grau@tuebingen.mpg.de**

Supplementary Figures

Experiment: Gain vs losses

In order to test if there exists a framing effect depending if the payoffs in the experiments are changed from losses to gains we conducted a second experiment. In this second experiment (reward vs. force payoff), sixteen subjects performed the urn and motor task experiment as described above. The only difference was the payoff mode. Subjects did not experience any viscous forces, but instead received point rewards. In urn task trials, a point was awarded whenever a red ball was drawn from the urn selected by the subject. In motor task trials, a point was awarded whenever the subject managed to hit the target. In all other cases no points were awarded. The total point score was displayed on the screen at all times. Even though we found stronger significance in the urn task and weaker significance in the motor task for specific ambiguity levels, overall we found that our results were not significantly affected when comparing the subject population receiving force payoff to the subject population receiving point payoffs ($p > 0.15$, Wilcoxon ranksum test for each ambiguity condition in the urn task and $p > 0.5$, Wilcoxon ranksum test for each ambiguity condition in the motor task). The aggregate choice probabilities and model fits are shown in Fig. SA. This suggests, in line with a previous study [1], that ambiguity attitude is not sensitive to positive or negative payoff.

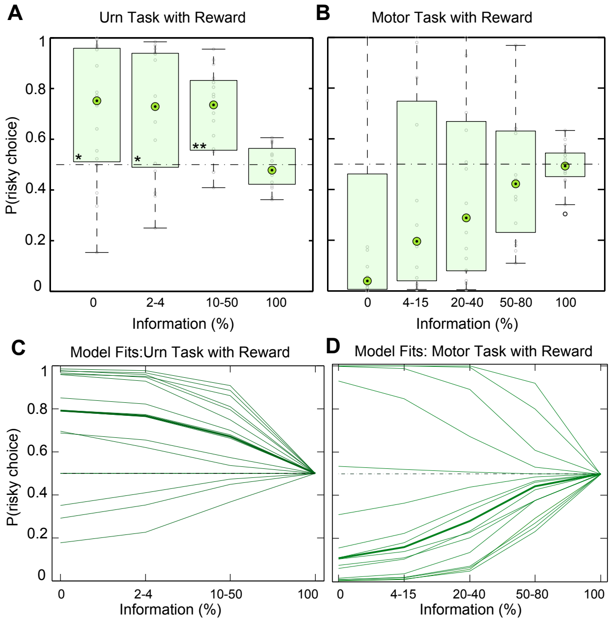

Figure A: **Experiment: Reward versus force payoff**. Aggregate choice probabilities over all subjects in symmetric probe trials of the urn task **A** and the motor task **B**, when subjects received point rewards instead of experiencing viscous forces. In the urn task, subjects received one point whenever a red ball was drawn from the urn the subjects selected. No points were awarded if a blue ball was drawn. In the motor task, subjects received one point for hitting the target, otherwise no point was awarded. In both tasks the total point score was shown at all times on the screen. The boxes are centered around the median across subjects and the edges of the box are the 25th and 75th percentiles. Panels **C** and **D** show the corresponding model fits. The thin green lines represent individual subjects' choice probabilities according to Equation (4) in the main text, the thick green line indicates the group mean. The dashed lines show the indifference choice probabilities predicted by expected utility. Probabilities above the dashed line imply that subjects prefer the risky choice (ambiguity aversion), probability values below the dashed line imply that subjects prefer the ambiguous choice (ambiguity preference). Asterisks denote significant deviation from the expected utility prediction: one asterisk signifies $p < 0.05$, two asterisks signify $p < 0.01$. In the urn task information (%) corresponds to the ratio of the number of revealed balls to the total number of balls, in the motor task to the ratio of visible size to total size of the ambiguous target.

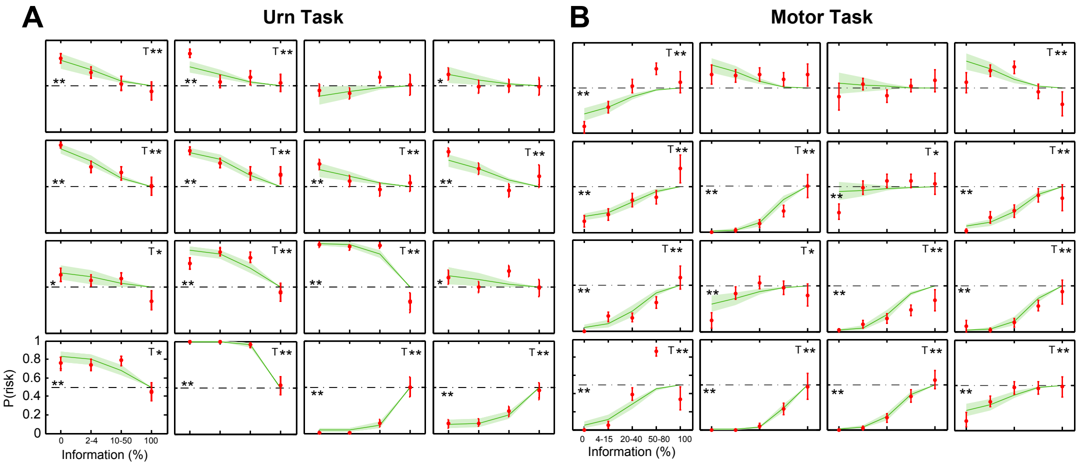

Figure B: **Experiment 1: Individual choice probabilities in probe trials.** in the urn task **(A)** and in the motor task **(B)**. The red data points show subjects' probability of choosing the risky option in dependence of the amount of information revealed from the ambiguous option. The error bars indicate 80% confidence intervals. In probe trials, an expected utility decision-maker should always be indifferent between the two options, independent of the information (dashed lines). The shaded green line shows maximum likelihood model fits for subjects' choice probability according to Equation (2) in the main manuscript. Asterisks on the first data point in each panel denote a significant difference from the dashed expected utility line. Asterisks in the top right corner of each panel indicate significance of trend. One asterisk signifies $p < 0.05$, two asterisks signify $p < 0.01$. In the urn task information (%) corresponds to the ratio of the number of revealed balls to the total number of balls, in the motor task to the ratio of visible size to total size of the ambiguous target. In total there were 16 subjects performing the two tasks. Each subject can be identified by their panel position.

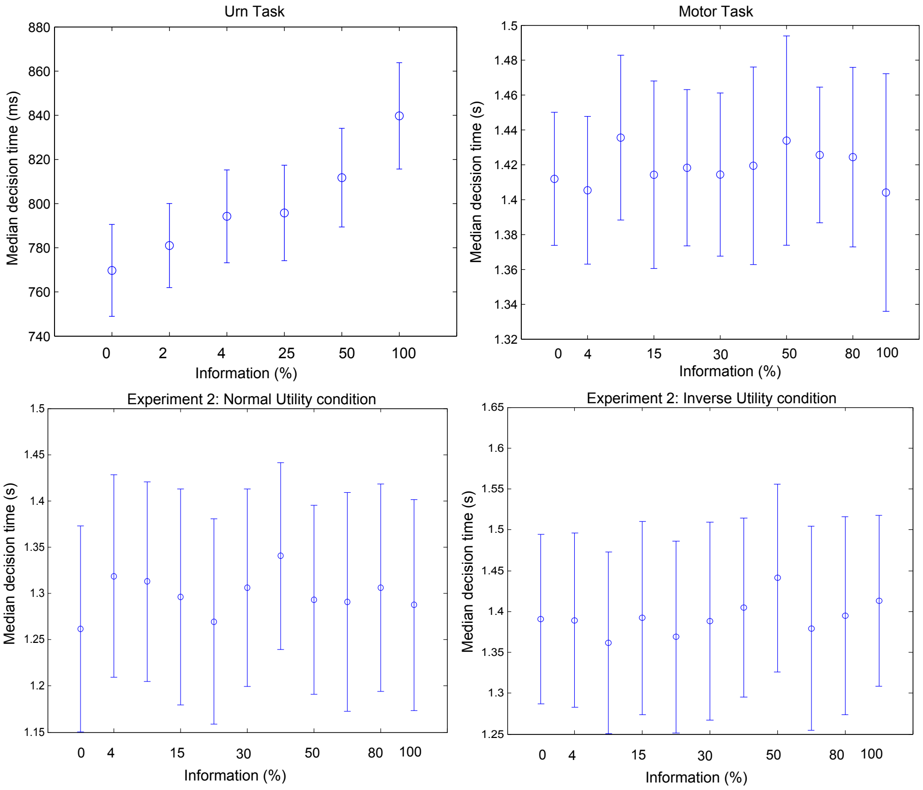

Figure C: **Average decision time across subjects in dependence of the amount of information revealed:** in the urn task (top-left panel), the motor task (top-right panel) and the control tasks of Experiment 2 (bottom-left and bottom-right panel). Error bars indicate standard errors. In the urn task, the decision time was defined as the time from entering the grey start bar to crossing into the orange zone displayed in Fig. 1 of the main text. In the motor task and Experiment 2, the decision time was defined as the time from entering the red square to entering one of the decision circles. Note that the decision time in the urn task was recorded for all 16 subjects, but in the motor task only for the last 8 subjects, and in Experiment 2 for all 25 subjects.

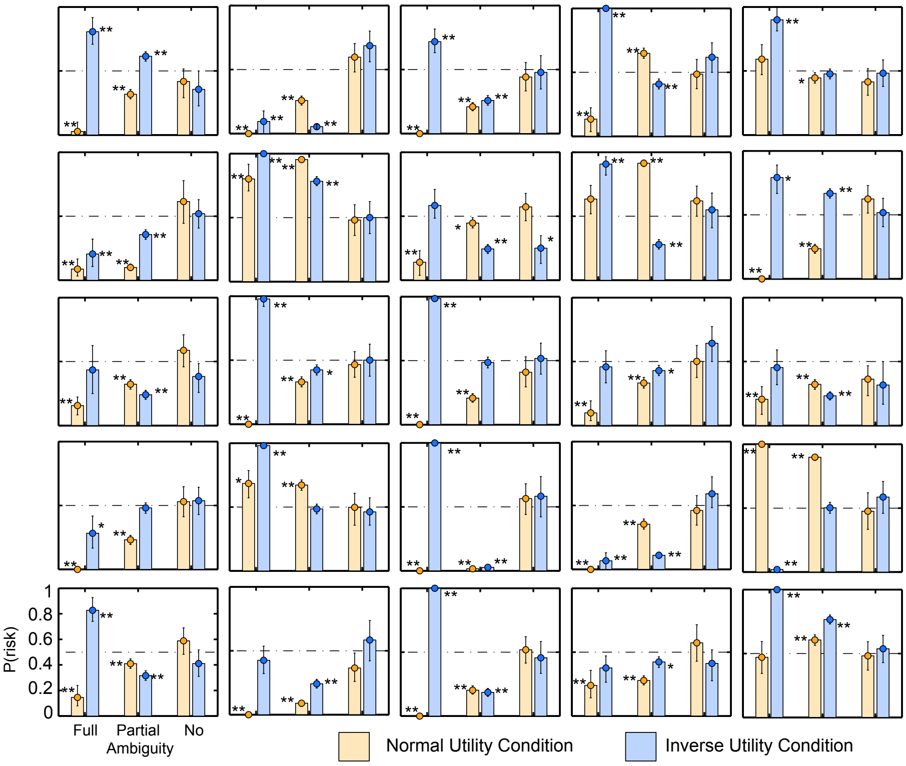

Figure D: **Choice probabilities of control experiment.** Individual choice probabilities in probe trials of the same subjects performing in the inverse utility **(A)** and the normal utility condition **(B)** under full, partial and no ambiguity. The data points show subjects' probability of choosing the risky option in dependence of the amount of information revealed from the ambiguous option. In probe trials, an expected utility decision-maker should always be indifferent between the two options (dashed lines). In 25 subjects, 11 subjects changed from general ambiguity preference in the normal utility condition to a mixed behavior in the inverse utility condition as reflected in the population average shown in the main manuscript. These subjects maintain ambiguity preference for partially ambiguous target bars, but become ambiguity averse in the full ambiguity condition. Six subjects maintained their ambiguity preference across utility conditions in line with the hypothesis that the stimulus induces a stable ambiguity attitude across all ambiguity conditions. Four subjects switched their ambiguity preference across utility conditions in line with a biased belief or perceptual distortion hypothesis. Asterisks on the data points denote a significant deviation from the dashed expected utility line. One asterisk signifies $p < 0.05$, two asterisks signify $p < 0.01$.

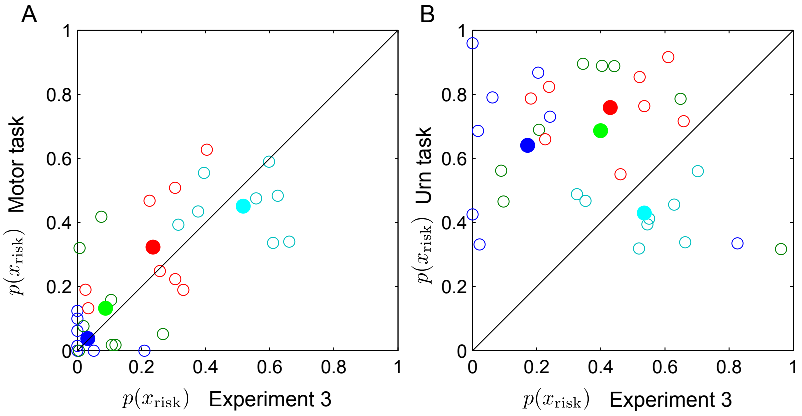

Figure E: **Comparison between choice probabilities**. Comparison between choice probabilities in Experiment 3 against choice probabilities in the motor task (**(A)**, 8 subjects, first group) and in the urn task (**(B)**, 8 subjects, second group)—see Methods. Each open circle corresponds to a subject's choice probability in one of the ambiguity conditions. The different colors indicate the ambiguity condition ranging from cyan, red, green and blue to denote the range from zero ambiguity to full ambiguity. Data points close to the diagonal line imply that the ambiguity preference of subjects remains stable across tasks (as in **(A)**), data points far from the diagonal line indicate that ambiguity attitudes of subjects changed (as in **(B)**), thus meaning that Experiment 3 and the motor task induced similar ambiguity attitudes. Filled circles denote the average across individual data points for each ambiguity condition.

References

[1] Keigo Inukai and Taiki Takahashi. Decision under ambiguity: effects of sign and magnitude. *International Journal of Neuroscience*, 119(8):1170–1178, 2009.
